# The use of ketogenic diets in cancer patients: a systematic review

**DOI:** 10.1007/s10238-021-00710-2

**Published:** 2021-04-03

**Authors:** Maximilian Römer, Jennifer Dörfler, Jutta Huebner

**Affiliations:** Klinik Für Innere Medizin II, Hämatologie Und Internistische Onkologie, Universitätsklinikum Jena, Am Klinikum 1, 07747 Jena, Germany

**Keywords:** Humans, Metabolism, Ketogenic diet, Low-carbohydrate diet, Carbohydrate-restricted, Cancer

## Abstract

Ketogenic diets are a widely known, yet controversial treatment for cancer patients. In this review, we summarize the clinical evidence for anti-tumor effects, as well as the effects on anthropometry, quality of life, adverse events and adherence in cancer patients. In April 2019, a systematic search was conducted searching five electronic databases (EMBASE, Cochrane, PsychInfo, CINAHL and Medline) to find studies analyzing the use, effectiveness and potential harm of a ketogenic diet in cancer patients of any age as sole or complementary therapy. From all 19.211 search results, 46 publications concerning 39 studies with 770 patients were included in this systematic review. The therapy concepts included all forms of diets with reduced carbohydrate intake, that aimed to achieve ketosis for patients with different types of cancer. Most studies had a low quality, high risk of bias and were highly heterogeneous. There was no conclusive evidence for anti-tumor effects or improved OS. The majority of patients had significant weight loss and mild to moderate side effects. Adherence to the diet was rather low in most studies. Due to the very heterogeneous results and methodological limitations of the included studies, clinical evidence for the effectiveness of ketogenic diets in cancer patients is still lacking.

**Supplementary Information:**

The online version contains supplementary material available at 10.1007/s10238-021-00710-2.

## Introduction

Current cancer treatment is largely based on surgery, radiation and chemotherapy. Despite the advances in these fields and the implementation of targeted therapies and immune checkpoint inhibitors, many cancer patients still suffer from a poor prognosis and search for alternative or complementary treatments. Since there is a growing recognition of the impact of dietary interventions on human health [1], many cancer patients try to optimize their diet to improve their prognosis and reduce treatment-associated side effects [2].

For these patients and professionals alike, the ketogenic diet (KD) is compelling due to its success in treating epilepsy [3] and its theoretical foundation. The proposed anti-tumor effect relies on Warburg’s observation, that cancer cells prefer anaerobic glycolysis, even in the presence of oxygen [4]. Further, cancer cells use glycolysis for rapid cell proliferation [5] and the formation of metastases [6]. Hence, KDs, which are high in fat and low in carbohydrates [7], try to reduce the amount of glucose in the body, that the cancer cells can utilize [8, 9]. The exact ratio of macronutrients differs between the specific variations of this diet [10]. Probably, the most renown adaption of this diet is a 4:1 fat-to-carbohydrate + protein ratio [7]. Such an approach was used successfully in cellular and animal studies [11, 12]. Nonetheless, there were also contradicting studies that showed that there are cancer cell lines, which can utilize fatty acids and ketone bodies [13–16].

Our aim in this review was to systematically assess whether the results from in vitro studies translated to clinical evidence of anti-tumor efficiency and further analyze the impact that a KD has on the quality of life and anthropometry of the patients.

## Method

### Criteria for including and excluding studies in the review

Inclusion and exclusion criteria are listed in Table 1 based on a PICO model. According to the recommendations of the Cochrane Effective Practice and Organization of Care (EPOC) systematic reviews, review and meta-analyses, randomized controlled studies (RCT), non-randomized controlled studies (CT), uncontrolled studies (process monitoring, uncontrolled before–after studies and time series analyses) and observational studies were included [17]. We additionally included case series and case studies, due to the low number of publications on this topic. Criteria for rejecting studies were primary prevention, gray literature, other publication types than primary investigation/report (e.g., comments, letters, abstracts) or precancerous conditions if the results of the patients with cancer were not reported separately. Additionally, studies were excluded if they reported no patient-centered outcomes (laboratory parameters, except PSA which was considered as a surrogate parameter for tumor progression of prostate cancer). Language restrictions were made to English and German.

**Table 1:** Inclusion and exclusion criteria based on a PICO model

PICO	Inclusion criteria	Exclusion criteria
Patient	Cancer patients (all entities and stages)	Patients with precancerous conditions or carcinoma in situ Primary prevention Preclinical studies
Intervention	Every intervention based on a ketogenic diet No restrictions regarding the type of KD, dose, mode of application KD applied as sole or supplementary treatment	
Comparison	All possible control groups (active control, placebo, standard/guideline/usual care)	
Outcome	Mortality (overall survival) Morbidity (progression-/disease-free interval, tumor response) Patient-reported outcomes (i.e., quality of life or other important psychological outcomes like psychological well-being, fatigue, as well as physical and mental adverse effects) Weight and body composition Toxicity and adverse events (CTCAE)	
Others	Language: German and English Full publication	Gray literature (conference articles, abstracts, letters, ongoing studies, unpublished literature, etc.) Full text not available in German or English

### Study selection

**Fig. 1:**
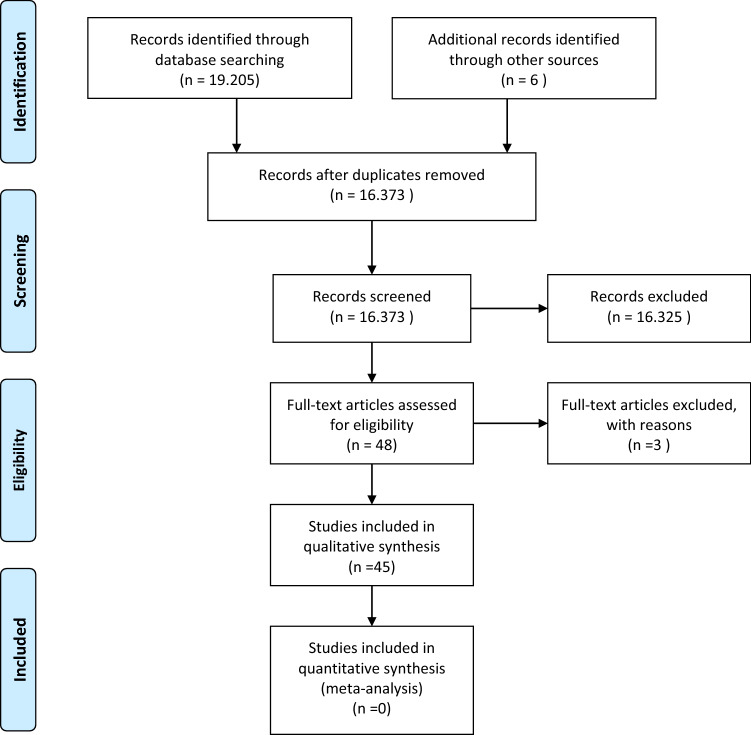
Preferred reporting items for systematic reviews and meta-analyses flow chart displaying the study selection process

A systematic research was conducted using five databases (Medline (Ovid), CINAHL (EBSCO), EMBASE (Ovid), Cochrane CENTRAL and PsycINFO (EBSCO)) in April 2019. For each of these databases, a complex search strategy was developed, consisting of a combination of MeshTerms, keywords and text words in different spellings connected to cancer and ketogenic diets. The detailed search string is provided in online resource 1. The search string was highly sensitive, since it was largely unrestricted by filters for study or publication type. After importing the search results into EndNote X9, all duplicates were removed and a title–abstract screening was carried out by two independent reviewers (MR, JD). In case of disagreement, consensus was made by discussion. After that, all full texts were retrieved and screened again independently by both reviewers. When title and abstract did not have sufficient information for screening purposes, a full-text copy was retrieved as well. Additionally, bibliography lists of all retrieved articles were manually screened for relevant studies. Such studies were included if they provided a comprehensive description of the study. The study flow during this process is presented in Fig. 1.

### Assessment of risk of bias and methodological quality

All characteristics were assessed by two independent reviewers (MR, JD). In case of disagreement, a third reviewer was consulted (JH) and consensus was made by discussion.

#### Risk of bias and methodological quality

The risk of bias of the included RCTs and CTs was analyzed with the SIGN-Checklist [18] for controlled trials version 2.0. The AMSTAR-2 instrument for systematic reviews was used for reviews and systematic reviews. Other study types were analyzed based on the Cochrane Risk of Bias tool [19]. Further, these studies were rated with the Oxford criteria. Additional criteria concerning methodology were size of population, application of power analysis, adequacy of statistical tests (e.g., control of premises or multiple testing) and selective outcome reporting (report of all assessed outcomes with specification of statistical data as the *p*-value) as well as possible conflicts of interest.

#### Data synthesis

No studies were suitable for a pooled analysis; hence, only a narrative analysis can be presented here.

### Data extraction

Data extraction was performed by one reviewer (MR) and controlled by two independent reviewers (JD, JH). As a template for data extraction, the evidence tables from the national Guideline on Complementary and Alternative Medicine in Oncological Patients of the German Guideline Program in Oncology (https://www.leitlinienprogramm-onkologie.de/english-language/) were used. Due to a large overlap in studies included in the systematic reviews, only the data from the individual studies, which were included in the systematic reviews, were extracted. Extraction was limited to data from primary literature and other sources, which provided a comprehensive description of the study, meeting the inclusion criteria.

## Results

The systematic search revealed 19.205 results. Six studies were added by hand search. At first, duplicates were removed leaving 16.373 studies. After screening title and abstract, 48 studies remained to complete review. Finally, 45 publications were analyzed in this review, including 5 SR, 1 review and additionally 5 publications on 3 RCTs, 2 controlled studies and 33 single-arm studies and case reports, presented in 32 publications. All in all, 765 patients were described in 39 publications.

### Characteristics of included studies

Concerning the RCTs and CTs, 322 patients were included and 250 of them were analyzed, due to 72 drop-outs. The mean age of patients (only reported in 3 studies) ranged from 44.8 to 66.3 years and the range of age from 38 to 76 years (reported in 1 study). One publication only reported the median age, which was 72 years. A total of 156 (62%) participants were female and 94 (38%) were male. Concerning the studies with a fixed duration of intervention, the extent of the diet ranged from 3 to 6 months.

In the single-arm studies and case series, a total of 443 patients were included and analyzed. The age of the included patients ranged from 3 to 92 years. Information about the gender of the included patients could be obtained for 370 of the 443 patients. Out of these 370 participants, 184 (50%) were female and 186 (50%) were male. Duration of the dietary intervention in these studies reached from a single, 3 h long, application of parenteral nutrition [20] to a single case with more than 31 months of oral KD [21].

The KDs prescribed in the included studies varied extensively between studies (see Table 6 for details concerning the prescribed diets) were in most articles not described in adequate detail, and the majority did not utilize standardized dietary protocols. Furthermore, the methods used for assessing ketone body levels and diet compliance varied widely, with some studies not measuring them at all.

### Excluded studies

A list of the studies excluded after full-text screening and the reasons for exclusion are presented in online resource 2.

### Risk of bias in included studies

The methodical quality of the included RCTs and CTs was rated according to the SIGN checklists [18], and the results are presented in Table 2. Other study types were analyzed based on the Cochrane Risk of Bias tool [22], with the results presented in Table 3. These studies were further rated based on the Oxford criteria. These results and additional comments on methodology are provided in Table 4.

**Table 2:** Risk of bias in the included RCTs and CTs according to the SIGN checklist

Reference	Study type	Standardized rating of risk of bias	Additional comments on methodology	Evidence level (Oxford)
Freedland et al. [24]	RCT	Rating according to SIGN Positive: 4 points Uncertain: 2 points Negative: 3 points Overall quality: acceptable	PRO: in accordance with the ethical guidelines of the US Common Rule; randomization stratified by center and BMI; compliance surveilled in arm A with weekly urine ketone measurement; comprehensive and adequate analysis including the most important factors; power analysis Contra: small sample size; no possibility for separation of the effects of weight loss and carbohydrate deficit; no information about approval by the ethics committee; no intention to treat analysis	1b–
Khodabakhshi et al. [23]	RCT	Rating according to SIGN Positive: 3 points Uncertain: 3 points Negative: 2 points Overall quality: acceptable	PRO: study protocol approved by responsible research institute; power analysis; groups are comparable Contra: small sample size, especially concerning the subgroup of neoadjuvant-treated patients; no survival analysis for the whole study population, only for subgroup of neoadjuvant-treated patients; unclear if intention to tread analysis was actually performed, since only information about patients that completed the study is given; duration of follow-up for survival analysis is longer than timeframe from start of patient enrollment to submission of the article	2b–
Cohen et al. [32, 42, 56]	RCT	Rating according to SIGN Positive: 4 points Uncertain: 2 points Negative: 3 points Overall quality: acceptable	PRO: compliance surveilled in arm A with weekly urine ketone measurement; inclusion of the demographic characteristics as covariates during analysis; power analysis; groups are comparable; study approved by the local institutional review board Contra: no adjustment for multiple testing; no intention to treat analysis; high drop-out; possible side effects are not mentioned	2b
Klement et al. [33]	CT	Rating according to SIGN Positive: 3 points Uncertain: 1 point Negative: 0 points Overall quality: acceptable	PRO: compliance surveilled in arm A with weekly beta-hydroxybutyrate blood measurement and patient questioning; analysis including the most important factors using linear mixed effects model; groups are comparable; study approved by the ethics committee Contra: small sample size, especially in arm A; no possibility for separation of the effects of ketogenic diet and amino acid supplementation; low objectively measured diet adherence using blood beta-hydroxybutyrate levels (69%)	3b
Ok et al. [34]	CT	Rating according to SIGN Positive: 3 points Uncertain: 0 points Negative: 1 point Overall quality: acceptable	PRO: study protocol approved by responsible institutional review board; groups are comparable Contra: small sample size; no possibility for separation of the effects of carbohydrate deficit and smaller but more frequent servings per day in intervention group; no power analysis; high drop-out; short follow-up	3b

RCT: randomized controlled trial; CT: non-randomized controlled trial; SIGN checklist: Scottish Intercollegiate Guidelines Network Methodology: Checklist 2: Randomized Controlled Trials

**Table 3:** Risk of bias in the included single-arm studies and case reports according to the Cochrane risk of bias tool

Study	RSQ	AC	BPP	BOA	IOD	SR	OSB
Champ et al. [45]	−	−	−	−	+	+	+
Fearon et al. [44]	−	−	−	−	+	+	+
Fine et al. [31]	−	−	−	−	+	+	+
Jansen and Walach [54]	−	−	−	−	+	+	−
Klement and Sweeney [41]	−	−	−	−	+	+	+
Martin-McGill et al. [35]	−	−	−	−	−	+	−
Martin-McGill et al. [49]	−	−	−	−	+	+	−
Rieger et al. [30]	−	−	−	−	+	+	−
Schmidt et al. [36]	−	−	−	−	−	+	+
Tan-Shalaby et al. [37]	−	−	−	−	−	+	+
van der Louw et al. [29]	−	−	−	−	−	+	+
Woodhouse et al. [28]	−	−	−	?	+	+	+
Zahra et al. KETOLUNG [38]	−	−	−	−	−	+	+
Zahra et al. KETOPAN [38]	−	−	−	−	−	+	+
Bozzetti et al. [48]	−	−	−	−	+	+	+
Schwartz et al. [39]	−	−	−	−	+	+	+
Zuccoli et al. [46]	−	−	−	−	+	+	+
Tóth and Clemens [47]	−	−	−	−	+	+	+
Bozzetti et al. [20]	−	−	−	−	+	+	+
Branca et al. [57]	−	−	−	−	+	+	+
Nebeling et al. [40]	−	−	−	−	+	+	+
Rossi-Fanelli et al. [43]	−	−	−	−	+	+	+
Schroeder et al. [58]	−	−	−	−	+	+	+
Artzi et al. [21]	−	−	−	−	+	+	+
Iyikesici [26]	−	−	−	−	+	+	+
Iyikesici [27]	−	−	−	−	+	+	+
Strowd et al. [25]	−	−	−	−	+	+	+
Moore [50]	−	−	−	−	+	+	−
Elsakka et al. [59]	−	−	−	−	+	+	+
Schwalb et al. [55]	−	−	−	−	+	+	−
Brünings [60]	−	−	−	−	?	?	?
Brünings [61]	−	−	−	−	?	?	?
Schütz [62]	−	−	−	−	?	?	?

**Table 4:** Risk of bias in the included single-arm studies and case reports rated with the Oxford criteria

Reference	Study type	Standardized rating of risk of bias	Additional comments on methodology	Evidence level (Oxford)
Champ et al. [45]	Retrospective single-arm clinical study	–	PRO: study approved by responsible institutional review board, adherence checked with urine and blood ketone bodies measurements Contra: small sample size, no standardized KD, no possibility for separation of the side effects caused by KD and concurring radio-chemotherapy	4
Fearon et al. [44]	Crossover study	–	PRO: study approved by local hospital ethical committee, crossover design to minimize confounding by covariates Contra: small sample size, no wash-out period resulting in possible carryover effects, extremely short duration of intervention	4
Fine et al. [31]	Prospective single-arm pilot study	–	PRO: study approved by responsible committee on clinical investigations, adherence checked with written food-recall records and blood ketone bodies measurements Contra: small sample size, no standardized KD, no possibility for separation of the effects caused by KD and weight loss	4
Jansen and Walach [54]	Systematic, prospective cohort study	–	PRO: Contra: small sample size, number of observations for the majority of the variables reported insufficient to perform a reliable statistical analysis; no standardized KD, no information about an approval by the responsible ethics committee; no information about the occurrence of side effects; potential conflict of interest: the first author is a shareholder of the company, that patients were specifically informed about as a source for ketogenic food	4
Klement and Sweeney [41]	Prospective Case reports	–	PRO: study approved by institutional ethics review board, adherence checked with food diaries written by the patients and monitoring of ketone levels in urine and blood Contra: very small sample size, no standardized KD, no possibility for separation of the effects caused by KD and radio(chemo)therapy	4
Martin-McGill et al. [35]	Randomized, mixed methods, feasibility study	–	PRO: study approved by local research ethics committee, adherence checked with food diaries written by the patients and monitoring of ketone levels in urine and blood; randomized Contra: small sample size, potential conflict of interest: the first author received a PhD studentship of the company, that provided the medium-chain triglyceride nutritional products used in Arm A; two co-authors received salary costs from the same company	4
Martin-McGill et al. [49]	Prospective single-arm pilot study	–	PRO: study approved by local Research, Development and Innovation committee; adherence checked with food diaries written by the patients and monitoring of ketone levels in urine Contra: very small sample size, potential conflict of interest: the first author received a PhD studentship from a company, that produces KD foods and supplements	4
Rieger et al. [30]	Prospective single-arm pilot study	–	PRO: study approved by local institutional review boards of the participating hospitals; adherence checked with nutritional questionnaires and monitoring of ketone levels in urine Contra: relatively small sample size, potential conflict of interest: one of the co-authors is the founder of a company, that produces KD foods and supplements and provided the nutritional packages used in the study; data not stratified by center	4
Schmidt et al. [36]	Prospective, single-arm pilot study	–	PRO: study approved by local ethics committee; adherence checked with patient documenting food intake and monitoring of ketone levels in urine Contra: small sample size, no standardization of KD despite carbohydrate intake	4
Tan-Shalaby et al. [37]	Single-arm prospective feasibility trial	–	PRO: study approved by local Independent Review Board Contra: small sample size, no standardized KD; no possibility for separation of the effects caused by KD and weight loss	4
van der Louw et al. [29]	Prospective single-arm feasibility study	–	PRO: study approved by local medical ethical committee; adherence checked with monitoring of the ketone body levels in the blood Contra: small sample size, no possibility for separation of the effects caused by KD and radio-chemotherapy	4
Woodhouse et al. [28]	Retrospective single-arm feasibility study	–	PRO: study approved by local institutional review board; adherence checked with monitoring of the ketone body levels in the blood Contra: small sample size, no possibility for separation of the effects caused by KD and radio-chemotherapy; retrospective study that only includes patients who achieved ketosis	4
Zahra et al. KETOLUNG [38]	Prospective single-arm phase 1 clinical trial	–	PRO: study approved by local institutional review board; adherence checked with food diaries written by the patients and monitoring of the ketone body levels in the blood Contra: small sample size, no possibility for separation of the effects caused by KD and radio-chemotherapy	4
Zahra et al. KETOPAN [38]	Prospective single-arm phase 1 clinical trial	–	PRO: study approved by local institutional review board; adherence checked with food diaries written by the patients and monitoring of the ketone body levels in the blood Contra: small sample size, no possibility for separation of the effects caused by KD and radio-chemotherapy	4
Bozzetti et al. [48]	Single case report	–	PRO: adherence secured, due to parenteral feeding CONTRA: only a single patient analyzed	4
Schwartz et al. [39]	Case Report	–	PRO: study approved by local institutional review board; adherence checked with monitoring of the ketone body levels in the blood CONTRA: extremely small sample size	4
Zuccoli et al. [46]	Case Report	–	PRO: CONTRA: only a single patient analyzed; no possibility for separation of the effects caused by KD and radio-chemotherapy; no systematic assessment of adverse effects	4
Tóth and Clemens [47]	Case report	–	PRO: adherence checked with monitoring of ketone levels in urine CONTRA: only a single patient analyzed; no possibility for separation of the effects caused by KD and radiotherapy; no systematic assessment of adverse effects; no standardized KD	4
Bozzetti et al. [20]	Single-arm prospective Study	–	PRO: power analysis Contra: small sample size; due to dietary intervention of only 3-h results can hardly be translated to the effects of a long-term dietary intervention	4
Branca et al. [57]	Single case report	–	PRO: Contra: only a single patient analyzed; no assessment of adverse effects	4
Nebeling et al. [40]	Case reports	–	PRO: study approved by local institutional review board; adherence checked with food diaries and monitoring of the ketone body levels in blood and urine Contra: small sample size; no systematic assessment of adverse effects; no possibility for separation of the effects caused by KD and radio-chemotherapy	4
Rossi-Fanelli et al. [43]	3-Arm prospective Study	–	PRO: adherence secured, due to parenteral feeding Contra: no assessment of adverse effects	3b
Schroeder et al. [58]	Prospective quantitative study	–	PRO: study approved by local research ethics committee; prospective study Contra: no assessment of adverse effects; no standardized diet; due to dietary intervention lasting only 4 days at most, results can hardly be translated to the effects of a long-term dietary intervention	4
Artzi et al. [21]	Prospective,2 arm pilot study	–	PRO: study approved by local institutional review board; adherence checked with monitoring of the ketone body levels in the urine Contra: no assessment of adverse effects; small sample size; control group added retrospectively	4
Iyikesici [26]	Single-arm retrospective study	–	PRO: due to the retrospective nature no institutional review board approval required Contra: no standardized diet; no possibility for separation of the effects caused by the KD and the additional treatments, including: polychemotherapy and hyperthermia	4
Iyikesici [27]	Single-arm retrospective study	–	PRO: due to the retrospective nature no institutional review board approval required Contra: no standardized diet; no possibility for separation of the effects caused by the KD and the additional treatments, including: polychemotherapy, hyperbaric oxygen therapy and hyperthermia	4
Strowd et al. [25]	Single-arm study	–	PRO: study approved by institutional review board; adherence checked with monitoring of the ketone body levels in blood and urine Contra: no structured assessment of adverse effects; small sample size	4
Moore [50]	Single case report	–	PRO: Contra: no structured assessment of adverse effects; no possibility for separation of the effects caused by KD and chemotherapy	4
Elsakka et al. [59]	Single case report	–	PRO: study approved by institutional review board Contra: no structured assessment of adverse effects; no possibility for separation of the effects caused by KD and other treatments including surgery, radiation, chemotherapy and other novel treatments	4
Schwalb et al. [55]	Case reports	–	PRO: Contra: small sample size; no structured assessment of adverse effects; no possibility for separation of the effects caused by the KD and the additional novel treatments, including high dose vitamin D, colostrum and multiple food supplements; two of the authors own companies, which produced most of the food supplements used in this trial	4
Brünings [60]	Case reports	–	PRO: Contra: historic study, from a current standpoint outdated and often subjective methods used to assess the effects of the diet	4
Brünings [61]	Case reports	–	PRO: Contra: historic study, from a current standpoint outdated and often subjective methods used to assess the effects of the diet	4
Schulte and Schütz [62]	Case reports	–	PRO: Contra: historic study, from a current standpoint outdated and often subjective methods used to assess the effects of the diet	4

*KD* ketogenic diet

### Efficacy of the ketogenic diet

The study characteristics and all relevant results reported in the included RCTs and CTs are presented in Table 5. Similar information concerning the included single-arm studies and case reports is presented in Table 6.

**Table 5:** Study characteristics and outcomes reported in the included RCTs and CTs

References	Study type	*N*	Cancer site	Age	Intervention/duration	Endpoints	Outcomes
Freedland et al. [24]	RCT	Included patients *N* = 57 Analyzed patients *N* = 45 Arm A: *N* = 27 Arm B: *N* = 18 Drop-out Arm A: 4 Arm B:8	Prostate cancer	Median: 72y	Arm A: A low-carbohydrate diet, goal: (≤ 20 g per day), estimated actual carbohydrate intake: 37 g/day; supervision by dietitians by telephone weekly for the first 3 months and then every 2 weeks for the last 3 months Arm B: Control group (no dietary intervention) Duration: 6 months	1. PSADT 2. Weight loss 3. BMI 4. Waist circumference 5. Adverse events	1. **Per protocol,** no difference was found in log-transformed PSADT over the 6-months between arms using a T-test (mean values in LCD vs. control: 21 vs. 15 months, *p* = 0.446) **Post hoc exploratory** analyses of PSADT: after adjusting for key baseline covariates including baseline PSA, pre-study PSADT, treatment received (surgery vs. radiation) and accounting for hemoconcentration during the study, LCD significantly lowered log-transformed PSADT (28 vs 13 months, *p* = 0.021) 2. Significantly higher weight loss in Arm A, than in Arm B; Arm A pretest: 197.5 kg, Δ from baseline − 12.1 kg, Arm B pretest: 196.2 kg, Δ from baseline − 0.5 kg; between-group comparison at the end of the study *p* < 0.001 3. Significantly higher BMI reduction in Arm A, than in Arm B; Arm A pretest: 29.0 kg/m^2^, Δ from baseline − 3.9 kg/m^2^ Arm B pretest: 29.7 kg/m^2^, Δ from baseline-0.2 kg/m^2^; between-group comparison at the end of the study *p* < 0.001 4. Significantly higher waist circumference reduction in Arm A, than in Arm B; Arm A pretest: 107.0 cm, Δ from baseline − 11.8 cm Arm B pretest: 110.7 cm, Δ from baseline-0.5 cm; between-group comparison at the end of the study *p* < 0.001 5. Similar number of AEs at baseline in both groups; numerically more AEs in Arm A at 3 months (30 vs 19) and slightly more AEs in Arm A at 6 months (19 vs 15); only mild and one moderate AE (nausea) reported
Khodabakhshi et al. [23]	RCT	Included patients *N* = 77 Analyzed patients *N* = 60 Arm A: *N* = 30 Arm B: *N* = 30 Drop-out Arm A: 10 Arm B:7	Breast cancer	Intervention group: Mean: 44.8 years Control group: Mean:45.2 years	Arm A: Medium-chain triglycerides (MCT) based ketogenic diet (6% calories from Carbohydrates [CHO], 19% protein, 20% MCT, 55% fat); Patients received 500 ml of MCT oil from the Nutricia Company every 2 weeks Arm B: Standard Diet (55% CHO, 15% protein, and 30% fat) Duration: 3 months	1. Overall survival 2. Weight 3. BMI 4. Body fat	1. No data for the whole study population given; significantly prolonged survival in a subgroup of only neoadjuvant patients; log rank test for Kaplan–Meier *p* = 0.04 2.Significantly higher weight loss in Arm A, than in Arm B; Arm A pretest: 71.7 kg, Δ from baseline − 6.3 kg, Arm B pretest: 70.5 kg, Δ from baseline—1.3 kg; between-group comparison at the end of the study *p* < 0.001 3. Significantly higher BMI reduction in Arm A, than in Arm B; Arm A pretest: 28.47 kg/m^2^, Δ from baseline 2.57 kg/m^2^ Arm B pretest: 28.44 kg/m^2^, Δ from baseline-0.64 kg/m^2^; between-group comparison at the end of the study *p* < 0.001 4. Significantly higher reduction of body fat, adjusted for baseline value, in Arm A, than in Arm B; A pretest: 35.8%, Δ from baseline − 6.7%, Arm B pretest: 34.5%, Δ from baseline—3.7%; between-group comparison at the end of the study *p* = 0.03
Cohen et al. [32, 42, 56]	RCT	Included patients *N* = 73 Analyzed patients *N* = 45 Arm A: *N* = 25 Arm B: *N* = 20 Drop-out Arm A: 12 Arm B:16	Ovarian cancer, Endometrial cancer	Mean: 60.2 years	Arm A: Ketogenic diet (70% [≥ 125 g]: 25% [≤ 100 g]: 5% [< 20 g] energy per day from fat, protein, and carbohydrates) Arm B: American Cancer Society diet (ACS: high in fiber, low in fat) Individual diet advice from certified dietitians. Weekly e-mails or phone calls. One face-to-face meeting after baseline assessment Duration: 3 months	1. Physical and mental health status 2. Energy level 3. Hunger and satiety, and food cravings 4. Body composition	1. Significant between-group difference in PCS after adjusting for baseline values and chemotherapy status (*p* = 0.04), with fat loss added to the model, the effect was no longer significant (*p* = 0.064) 2. no significant between-group difference in MCS, only a subgroup of the participants in the intervention group without concurrent chemotherapy reported a statistically significant improvement of 23% in energy level from baseline to 12 weeks (*p* = 0.02) 3. significant less cravings for starchy foods and fast-food fats after adjusting for baseline values and chemotherapy status in the intervention group (*p* = 0.03 and *p* = 0.04, respectively) measured with FCI 4.significantly higher reduction of total body mass in Arm A, than in Arm B; Arm A pretest: 81.2 kg, Δ from baseline − 6.1 kg, Arm B pretest: 89 kg, Δ from baseline − 3 kg; between-group comparison at the end of the study *p* < 0.05 significantly higher reduction of total fat mass in Arm A, than in Arm B; Arm A pretest: 37.9 kg, Δ from baseline − 5.2 kg, *p* < 0.05, Arm B pretest: 44.1 kg, Δ from baseline − 2.9 kg, *p* > 0.05; between-group comparison at the end of the study *p* < 0.05 no significant differences in lean body mass between Arm A and Arm B; Arm A pretest: 43.2 kg, Δ from baseline − 0.9 kg, *p* > 0.05, Arm B pretest: 44.9 kg, Δ from baseline − 0.1 kg, *p* > 0.05; between-group comparison at the end of the study *p* > 0.05
Klement et al. [33]	Controlled study	Included patients *N* = 85 Analyzed patients *N* = 81 Arm A: *N* = 20 Arm B: *N* = 61 Drop-out Arm A: 2 Arm B:2	Rectal cancer, head and neck cancer Breast cancer	From 38 to 76 years	Arm A: ketogenic diet with additional consumption of non-glucogenic amino acids, patients are provided with literature regarding ketogenic diet; opportunity to speak with a dietician Arm B: control (no dietary intervention); in case of dietary counseling: official recommendations of the German Society for nutrition provided to the patient Duration: as long as the patients received RT (median duration: 35-40 days)	1. Diet adherence 2. Body composition changes	1. subjectively reported by patients: 100% objectively measured using blood BHB levels: 69% 2. Regression coefficients for body composition changes, according to the linear mixed-effects model. Effects of the KD over time were described with the coefficient “KD x Time” in the study: Rectal cancer patients: Regression coefficient for body weight change in Arm A compared to Arm B: − 0.4 kg/week, *p* = 0.011 Regression coefficient for fat-free mass change in Arm A compared to Arm B: 0.0 kg/week, *p* = 0.9467 Regression coefficient for fat mass change in Arm A compared to Arm B: − 0.5 kg/week, *p* = 0.000889 HNC patients: Regression coefficient for body weight change in Arm A compared to Arm B: + 0.6 kg/week, *p* = 0.00823 Regression coefficient for fat free mass change in Arm A compared to Arm B: + 0.4 kg/week, *p* = 0.03423 Regression coefficient for fat mass change in Arm A compared to Arm B: + 0.2 kg/week, *p* = 0.3296 breast cancer patients: Regression coefficient for body weight change in Arm A compared to Arm B: − 0.3 kg/week, *p* = 0.00124 Regression coefficient for fat free mass change in Arm A compared to Arm B: + 0.1 kg/week, *p* = 0.1655 Regression coefficient for fat mass change in Arm A compared to Arm B: − 0.4 kg/week, *p* = 8.49 × 10^–5^
Ok et al. [34]	Controlled study	Included patients *N* = 30 Analyzed patients *N* = 19 Arm A: *N* = 10 Arm B: *N* = 9 Drop-out Arm A: 10 Arm B:1	Pancreato-biliary cancer	intervention group: Mean: 57.8 years Control group: Mean: 66.3 years	Arm A: Ketogenic diet (3–6%, 14–27%; 70–80% energy per day from carbohydrates, protein, and fat) served as 3 meals and 3 snacks per day Arm B: usual Korean diet (55–65%, 7–20%, 15–30% energy per day from carbohydrates, protein and fat) served as 3 meals per day Duration: Measurement of meal compliance, energy and protein intake: 10 days Measurement of body composition and frequency of meal intake-related problems: till 1^st^ outpatient visit after surgery (mean hospital stay for Arm A = 12 days)	1. Average energy intake rate 2. Average protein intake rate 3. Frequency of meal intake-related problems 4. Body composition	1. Arm A:61.3%; Arm B: 38.5%; *p* < 0.05, significant higher in Arm A 2. Arm A:63.5%; Arm B:37.7%; *p* > 0.05, no significant difference 3. Arm A: average number of Problems per person 1.3 Arm B: average number of problems per person 2; *p* > 0.05, no significant difference 4. No significant differences in the reduction of body weight in Arm A, compared to Arm B; Arm A pretest: 64.6 kg, Δ from baseline − 4 kg, Arm B pretest: 56.2 kg, Δ from baseline − 3.5 kg; between-group comparison at the end of the study *p* = 0.475 significantly less reduction of body cell mass in Arm A, than in Arm B; Arm A pretest: 28.9 kg, Δ from baseline − 1.9 kg; Arm B pretest: 27.4 kg, Δ from baseline − 2.9 kg; between-group comparison at the end of the study *p* = 0.049; no significant differences in body fat mass between Arm A and Arm B; Arm A pretest: 18.2 kg, Δ from baseline − 1.1 kg Arm B pretest: 13.7 kg, Δ from baseline + 0.5 kg; between-group comparison at the end of the study *p* = 0.086

*PSADT* prostate-specific antigen doubling time, *AE* adverse event, *BMI* body mass index, *PCS* physical component summary, *MCS* mental component summary, *FCI* food craving inventory, *RT* radiotherapy, *BHB* beta-hydroxybutyrate, *HNC* head and neck cancer

**Table 6:** study characteristics and outcomes reported in the included single-arm studies and case reports

Reference	Study type	*N*	Cancer site	Age	Intervention/duration	Endpoints	Outcomes
Champ et al. [45]	Retrospective single-arm clinical study	Analyzed patients *N* = 53 Arm A: *N* = 6 Arm B: *N* = 47	Glioblastoma multiforme	From 34 to 62 years	Arm A: self-administered KD Arm B: unspecified standard American diet Duration: 3–12 months	1. Adverse events 2. Bodyweight	1. 2 patients with grade 1 constipation, 4 patients with grade 1 fatigue, 1 patient with grade 2 fatigue, 1 patient with deep venous thrombosis during treatment, 1 patient with asymptomatic hypoglycemia, 1 patient with nephrolithiasis no grade 3 and higher toxicities or symptomatic hypoglycemia 2. weight loss on non-calorie-restricted KD: 1 to 27Ibs Weight loss on calorie-restricted KD: 46Ibs
Fearon et al. [44]	Crossover study	Analyzed patients *N* = 5	Ovarian, Lung, Gastric	Mean: 61 years	Crossover study: Nasogastric tube feeding: normal, balanced regimen on days 1–6 KD containing same total calorie and protein on days 7–13 Duration: 13 days	1. Protein synthesis, turnover and nitrogen balance 2. Bodyweight 3. Performance status	1. No significant differences, mean daily *N* balance non-significantly more positive on normal, balanced diet, *p* > 0.1 2. No significant change in body weight during normal balanced diet, *p* > 0.05 Significant increase in body weight during KD (average + 2 kg), *p* < 0.05 3. Performance status did not change during normal balanced diet, but increased by one point during KD, but no testing for the statistical significance was applied
Fine et al. [31]	Prospective single-arm pilot study	Recruited patients *N* = 12 Analyzed patients *N* = 10	Diverse	Mean: 62.9 years	KD with targeted CHO intake below 5% of total energy intake, written menus and samples of CHO-restriction products were provided Duration: 28 days	1. Toxicity 2. Metabolic effects 3. Dietary adherence	1. 5 patients with grade 2 fatigue, 5 patients with grade 1 constipation, 1 patient with grade 1 leg cramps 2. Mean weight loss 4% compared to baseline, *p* = 0.08; all patients spontaneously decreased their caloric intake, mean energy deficit: 35%, *p* < 0.01 compared with baseline 3. 5 out of 12 patients completed all 28d of the diet
Jansen and Walach [54]	Systematic, prospective cohort study	Analyzed patients *N* = 78 Arm A: *N* = 7 Arm B: *N* = 6 Arm C *N* = 65	Diverse	Mean: 68.3 years	Arm A: full adoption of a non-specified KD, patients informed about a single company producing KD related food Arm B: partial adoption of a non-specified KD, patients informed about a single company producing KD related food Arm C: patients who did not adopt a KD Duration: non-specified, study began 11/2010, follow-up until end of 2011	1. TKTL 1 level 2. Improvement in cancer status	1. Reduction in TKTL 1 was associated with adopting a KD, no test for significance due to insufficient number of cases 2. Correlation between improvement in cancer status category and full adoption of a KD (χ2 = 33.26; df = 4; *p* = 0.00001), no information provided about the definitions and the exact methods used to define the cancer status categories
Klement and Sweeney [41]	Prospective Case reports	Analyzed patients *N* = 6	Diverse	From 40 to 74 years	Self-administered KD (recommended CHO intake < 50 g/day) during the course of RT/RCT; patients received basic information on KD; counseling at least once per week Duration: Patient dependent from 32 to 73 days	1. QoL 2. Bodyweight 3. Body composition	1. Only measured in 5 out of 6 patients, QoL at the end of RT decreased in 3 out of 5 patients and stayed consistent in 2 out of 5 2. Significant decrease in 2 patients, only analyzed individually, no analysis for the whole study population performed 3. Only 4 patients analyzed; FM decreased significantly in 3 patients, FFM did not change significantly
Martin-McGill et al. [35]	Randomized, mixed methods, feasibility study	Assessed for eligibility: *N* = 57 Randomized: *N* = 12 Arm A: *N* = 6 Arm B: *N* = 6 Retention at 12 weeks. *N* = 4 Arm A: *N* = 3 Arm B: *N* = 1	Glioblastoma	From 44 to 66 years	Arm A: MCTKD (75%; 15%; 10% of energy per day from fat, protein and carbohydrates, with 30% of fat from MCT nutritional products) Arm B: MKD (80%; 15%; 5% of energy per day from fat, protein and carbohydrates) Duration: 12 weeks	1. Long-term retention 2. Quality of life 3. Adverse events	1. Arm A: 3 patients retained for 3 months (drop-out = 50%) Arm B: 1 patient retained for 3 months (drop-out = 83%) 2. GHS at baseline: Arm A: patients who later withdrew: 72.2 ± 20.7; patients who retained: 75 ± 6.8 Arm B: patients who later withdrew: 70 ± 13.8; patients who retained: 80 ± 0 GHS: at week 6: Arm A: patients who withdrew at week 6: 41.7 ± 0; patients who retained: 66.7 ± 0 Arm B: patients who withdrew at week 6: 50 ± 0; patients who retained: 100 ± 0 3. Adverse events during the first 6 weeks: Arm A: diarrhea (*n* = 1, CTCAE grade 1), nausea (*n* = 1, CTCAE grade 1), vomiting (*n* = 1, CTCAE grade 2), dyspepsia (*n* = 1, CTCAE grade 1) Arm B: vomiting (*n* = 1, CTCAE grade 1), dry mouth (*n* = 1 MKD, CTCAE grade 1)
Martin-McGill et al. [49]	Prospective single-arm pilot study	Enrolled: *N* = 6 Completed intervention: *N* = 4	Glioblastoma	From 34 to 66 years	MKD (70%: 3–5% [≤ 20 g] energy per day from fat and carbohydrates; protein consumption was not restricted Duration: 12 weeks	1. Adverse events 2. Body composition	1. Constipation in 2 patients, resolved with dietary modification 2. No significant differences in body composition occurred
Rieger et al. [30]	Prospective single-arm pilot study	Included patients *N* = 20 Evaluable for efficiency *N* = 17	Glioblastoma	Median: 57 years	KD with CO intake < 60 g/day, additionally highly fermented yoghurt drinks and two different plant oils were provided to be consumed at will No calorie restriction, patients were instructed to always eat to satiety Duration: till progression of the disease	1. Feasibility 2. Bodyweight 3. Tolerability 4. Efficacy	1. 3 out of 20 patients discontinued the diet after 2–3 weeks without progression, due to reduced QoL 2. Significant body weight reduction; mean weight at baseline: 78.3 kg, mean weight at the end of the diet: 76.5 kg (*p* < 0.05) 3. Diarrhea, constipation, hunger and/or demand for glucose were present in a minority of patients during the diet 4. Median PFS on the KD alone was 5 weeks No significant difference between median PFS on the KD with additional bevacizumab treatment (20.1 weeks) and median PFS of patients on normal diet treated with bevacizumab in the same hospital during the same period (16.1 weeks) *p* = 0.38
Schmidt et al. [36]	Prospective, single-arm pilot study	Enrolled: *N* = 16 Completed intervention: *N* = 5	Diverse	From 33 to 64 years	KD with CHO limited to 70 g per day and 20 g per meal Two oil–protein shakes consumed in the morning and in the afternoon Duration: 12 weeks	1. Feasibility 2. Bodyweight 3. Adverse events 4. QoL	1. 11 out of 16 Patients discontinued the diet, 3 out of 11 were unable to adhere to the diet, 6 out of 11 discontinued due to progressive disease and 2 out of 11 died from progressive disease 2. Only analyzed in 7 patients; significant weight loss of 2 kg from mean 68.5 kg at baseline to 66.5 kg at the end of the diet, *p* < 0.05 3. Statistical evaluation of the adverse events and the influence on QOL is not statistically feasible; reported side effects included increase in appetite loss, constipation, diarrhea and fatigue during the diet 4. QoL was low at baseline and stayed relatively stable during the intervention; worsening of fatigue, pain, dyspnea and role function but emotional functioning and insomnia improved slightly
Tan-Shalaby et al. [37]	Single-arm prospective feasibility trial	Enrolled: *N* = 17 Drop-out before first analysis: *N* = 6 Completed intervention: *N* = 4	Diverse	From 42 to 87 years	Modified Atkins Diet with 20 to 40 g of CHO and restricted consumption of high CHO foods no restrictions for calories, protein or fats Duration 16 weeks	1. Feasibility 2. Bodyweight 3. Adverse effects 4. QoL	1. 13 out of 17 patients discontinued the diet before 16 weeks 2. Significant mean weight loss of all subjects: 7.5 kg, *p* < 0.05; significant mean weight loss of the patients, who completed the diet: 12.3 kg, *p* < 0.05 3. Reported adverse effects included: hyperuricemia (*N* = 7), hyperlipidemia (*N* = 2), pedal edema (*N* = 2), anemia (*N* = 2), halitosis (*N* = 2), pruritus (*N* = 2), hypoglycemia (*N* = 2), hyperkalemia (*N* = 2), hypokalemia (*N* = 2), hypomagnesemia (*N* = 2), flu-like symptoms/fatigue (*N* = 2) 4. Patients, who completed at least 4 weeks of the diet (*N* = 6) showed no significant deterioration in QoL
van der Louw et al. [29]	Prospective single-arm feasibility study	Eligible patients: *N* = 11 Included in phase A: *N* = 9 Included in phase B: *N* = 8 Completed intervention *N* = 6	Glioblastoma multiforme	Median: 53.8 years	Phase A: Fluid KD with a 4:1 ratio (4 g fat versus 1 g protein plus carbohydrates, 90% energy from fat) Patients were allowed a snack with the same 4:1 diet ratio once a day Phase B: Solid-food KD (diet ratio 1.5–2.0:1) with MCT; (70% energy from fat with the consistency of an emulsion) Duration: 14 weeks (6 weeks phase A, 8 weeks phase B)	1. Feasibility 2. Adverse effects 3. QoL 4. Overall survival	1. 6 out of 9 patients (67%) included in phase A completed the 14 weeks KD 2. Reported adverse effects included: CTCAE grade 1: constipation (*n* = 7), nausea/vomiting (*n* = 2), hypercholesterolemia (*n* = 1), hypoglycemia (*n* = 1), low carnitine (*n* = 1) and diarrhea (*n* = 1). CTCAE grade 2: hallucinations (*n* = 1), allergic reaction (*n* = 1) and wound infection (*n* = 1) 3. Global quality of live at baseline: 83%, global quality of live at end of study: 58%; reference value: 78% 4. The median overall survival of the nine patients was 12.8 months; median survival duration reference value is 15 months
Woodhouse et al. [28]	Retrospective single-arm feasibility study	Analyzed patients: *N* = 29	Glioma	From 30 to 76 y	MAD with a 0.8–1:1 ratio (0.8-1 g fat to 1 g carbohydrate plus protein Duration: 6 weeks	1. Feasibility 2. Adverse events 3. Changes in BMI	1. 28 out of 29 patients (96.6%) completed the 6-week diet 2. Grade 2 constipation (*n* = 1), grade 1 fatigue and nausea were present in the patients 3. Median change of BMI for all patients was –1.04 kg/m^2^, not analyzed for significance
Zahra et al. KETOLUNG [38]	Prospective single-arm phase 1 clinical trial	Screened patients: *N* = 11 Enrolled patients: *N* = 7 Completed intervention: *N* = 2	Lung	Median: completed KD: 66 years Did not complete: 67 years	KD with 90%; 8%; 2% of energy per day from fat, protein and carbohydrates. All meals readily prepared for the patients Duration: 42 days	1. Feasibility 2. Adverse events 3. Bodyweight	1. 2 out of 7 patients (29%) completed the intervention 2. Reported adverse events included: CTCAE Grade 1–2: constipation, diarrhea, nausea, vomiting and fatigue; 1 patient experienced DLT (hyperuricemia Grade 4) 3. Average weight loss: 5.6 kg
Zahra et al. KETOPAN [38]	Prospective single-arm phase 1 clinical trial	Screened patients: *N* = 5 Enrolled patients: *N* = 2 Completed intervention: *N* = 1	Pancreas	Completed KD: 69 years Did not complete KD: 67 years	KD with 90%; 8%; 2% of energy per day from fat, protein and carbohydrates. All meals readily prepared for the patients Duration: 34 days	1. feasibility 2. adverse events 3. bodyweight	1. 1 out of 2 patients (50%) completed the intervention 2. Reported adverse events included: CTCAE grade 1–2: Constipation, diarrhea, nausea and vomiting 1 patient experienced DLT (dehydration grade 3) 3. Average weight loss: 8.2 kg
Bozzetti et al. [48]	Single case report	*N* = 1	Desmoid tumor	28y	TPN consisting of 28 kcal fat/kg body weight/day, 1.5 g protein/kg body weight/day; 40 g glucose/day Duration: 5 months	1. bodyweight 2. adverse events	1. Body weight increased by 1 kg (from 61 to 62 kg) 2. No adverse events reported, no signs of hepatic steatosis or liver damage
Schwartz et al. [39]	Case report	Included patients: *N* = 2 Completed intervention: *N* = 1	Glioma	From 3 to 65 years	ERKD: with a 3:1 ratio of ingested nutrients (3 g fat versus 1 g protein plus carbohydrates) 20% restriction of calories per day Duration: 12 months	1. feasibility 2. adverse events 3. bodyweight	1. 1 out of 2 patients (50%) completed the intervention 2. Besides headaches no adverse events 3. Body weight initially decreased in both patients and remained stable afterward
Zuccoli et al. [46]	Case Report	*N* = 1	Glioblastoma multiforme	65 years	ERKD delivering 600 kcal per day, consisting of 42 g fat, 32 g protein and 10 g CHO per day Duration: 56 days	1. bodyweight 2. adverse events	1. bodyweight decreased 3 kg (from 58 to 55 kg) in the first 14 days of the diet 2. No adverse events despite grade 4 hyperuricemia reported, resulted in diet change to calorie restricted non-ketogenic diet
Tóth and Clemens [47]	Case report	*N* = 1	Rectal	62 years	Paleolithic KD, nutrients consumed in a fat: protein ratio of 2:1 animal fat, red meats and organ meats were encouraged, root vegetables were allowed, all other foods were prohibited Duration: 24 months	1. adverse events 2. bodyweight 3. tumor volume	1. No adverse events were reported 2. Bodyweight decreased 13 kg (from 78 to 65 kg) during the diet 3. Initial decrease in volume after concomitant radiotherapy; tumor volume remained stable in the following months, but four hepatic metastases were detected at the end of the diet
Bozzetti et al. [20]	Single-arm prospective Study	*N* = 12	Diverse	From 31 to 75 years	single 3 h infusion of glucose-based (GTPN) or a lipid-based TPN (LTPN) containing 4 mg glucose/kg/min or 2 mg lipid/kg/min, respectively Duration: 3 h	1. Glucose uptake analysis of the liver metastases using FDG-PET	1. No statistically significant stimulation or suppression of FDG uptake due to the administration of GTPN or LTPN
Branca et al. [57]	Single Case Report	*N* = 1	Breast	66 years	Self-administered high doses of oral vitamin D3 (10,000 IU/day), and KD rich in Oleic acid Duration: 3 weeks	1. changes in tumor biomarkers	1. Progesterone receptor status positivity increased from < 1% at baseline to 20% after the 3-week intervention; HER2 positivity decreased from > 10% (score 2 +) to 0% (score 0) after the 3-week intervention
Nebeling et al. [40]	Case reports	*N* = 2	Astrocytoma	From 3 to 8.5 years	KD with 60%; 20%; 10%, 10% of energy per day from MCT oil, protein, carbohydrates and dietary fat plus additional supplements Duration: 8 weeks	1. Glucose uptake analysis of the tumor using FDG-PET 2. feasibility	1. Dose uptake ratio tumor: normal cortex decreased by approximately 22% in both patients 2. 2 out of 2 (100%) patients were able to complete the dietary intervention
Rossi-Fanelli et al. [43]	3-Arm prospective Study	Enrolled: *N* = 27 Arm A: *N* = 9 Arm B: *N* = 9 Arm C: *N* = 9	Esophagus Stomach Colon–rectum	Median: Arm A: 61 years Arm B: 70 years Arm C: 67 years	Arm A: glucose-based TPN (100% of the calorie from dextrose) Arm B: lipid-based TPN (80% of the calorie from fat, 20% from dextrose) Arm C: oral diet All diets were iso-caloric and isonitrogenous Duration: 2 weeks	1. tumor cell kinetics 2. bodyweight	1. Assessed as the fraction of cells in S-phase; none of the changes within and between the three arms reached statistical significance 2. None of the changes within and between the three arms reached statistical significance
Schroeder et al. [58]	Prospective quantitative study	*N* = 12	Head and neck	From 50 to 86 y	Unspecified western diet followed by unspecified KD Duration: variable, up to 4 days	1. metabolic changes in the tumor tissue	1. Decline of mean lactate concentration in the tumor tissue during the KD, no analysis for statistical significance performed glucose and pyruvate concentration in the tumor tissue were stable or even increased, no analysis for statistical significance performed
Artzi et al. [21]	Prospective, two-arm pilot study	Included: *N* = 9 intervention: *N* = 5 retrospectively added control *N* = 4	Brain	From 27 to 69 years	KD based on ready-made formula, with a 4:1 ratio of ingested nutrients (4 g fat versus 1 g protein plus carbohydrates) Duration: variable from 2 to 31 months	1. feasibility 2. ketone body levels in the brain	1. Diet tolerated by 4/5 patients, strict adherence only in 2 patients 2. 4 out of 50 MRI spectroscopy scans detected ketone bodies in the brains of the patients following the KD None of the scans detected ketone bodies in the control group
Iyikesici [26]	Single-arm retrospective study	*N* = 44	Lung (NSCLC)	Median: 65 years	Mild KD (patients were encouraged to avoid high CHO food) in combination with HBO, hyperthermia and polychemotherapy administered during induced hypoglycemia Duration: 24 weeks Follow-up: 1–6 years	1. survival 2. adverse events	1. After 24 weeks 42 patients (95%) and at the termination of follow-up 29 patients (66%) were alive mean OS was 43 months (numerically better than historical controls from other studies) 2. Adverse events reported during treatment period: grade 5 neutropenia (*N* = 1), grade 3 neutropenia (*N* = 3), grade 3 anemia (*N* = 10), grade 4 thrombocytopenia (*N* = 3), grade 3 fatigue (*N* = 5), grade 3 diarrhea (*N* = 8), grade 3 neuropathy (*N* = 1), all of which were attributed to chemotherapy
Iyikesici [27]	Single-arm retrospective study	*N* = 25	Pancreas	Median: 61 years	Mild KD (patients were encouraged to avoid high CHO food) in combination with HBO, hyperthermia and polychemotherapy administered during induced hypoglycemia Duration: mean follow-up: 25 months	1. survival 2. adverse events	1. During follow-up mean OS was 15.8 months (numerically better than historical controls from other studies) 2. Adverse events reported during treatment period: grade 3/4 neutropenia (*N* = 9), febrile neutropenia (*N* = 1), grade 3 anemia (*N* = 7), grade 4 thrombocytopenia (*N* = 4), grade 3 diarrhea (*N* = 2), all of which were attributed to chemotherapy
Strowd et al. [25]	Single-arm study	Included *N* = 8 Completed intervention *N* = 7	Brain	From 28 to 54 years	MAD with20g CHO/ day restriction Duration: 2–24 months (mean 13.17 months)	1. Bodyweight 2. Seizure frequency	1. Non-significant body weight decrease by a mean 3.4 kg (*p* = 0.48) 2. Non-significant reduction in mean seizure frequency per week from 0.54 at baseline to 0.1 at 6 months (*p* = 0.27)
Moore [50]	Single case report	*N* = 1	Glioblastoma multiforme	40 years	Energy-restricted KD with a 4:1 ratio of calorie intake (fat versus protein plus carbohydrates) Total calories calculated 25% below BMR Duration: 4 months	1. Anti-tumor effect 2. Adverse events	1. PET-CT at the end of the diet detected no metabolically active tumor, despite a new enhancement area in MR 2. No significant fatigue or reduced mental capacity reported, patient was able to continue his work and exercise regime
Elsakka et al. [59]	Single case report	*N* = 1	Glioblastoma multiforme	38 years	KD with a 4:1 ratio of calorie intake (fat versus protein plus carbohydrates), delivered as calorie restricted diet, combined with intermittent fasting, HBOT, other novel therapies and SOC treatment Duration: 20 months	1. Anti-tumor effects 2. Body weight 3. QoL 4. Anti-tumor effect	1. Good surgical outcome and regressive changes in histopathology 2. Body weight decreased 9.3 kg during the intervention 3. No clinical or neurological symptoms reported, despite reduced weight no discomfort 4. After subtotal tumor resection, radio- and chemotherapy stationary disease
Schwalb et al. [55]	Case reports	*N* = 6	Diverse	From 55 to 73 years	Very low CHO diet (not further specified) with a multitude of supplements, including amino acids and Vitamin D^3^ combined with SOC therapy Duration: variable	1. anti-tumor effects 2. effect on cancer related symptoms	1. shrinkage of tumor or stable disease was reported during the intervention 2. subjective improvement reported in some cases
Brünings [60]	Case reports	*N* = 14	Head and neck		KD with as little CHO as possible (estimated < 50 g per day), combined with insulin administration 3 × per day	1. Anti-tumor effects 2. Adverse events	1. visible remission after 2–3 weeks, but rebound effect after 2–3 months on the diet 2. no adverse events were reported
Brünings [61]	Case reports	*N* = 30	Extra-cranial		KD with as little CHO as possible (estimated < 50 g per day), combined with insulin administration 3 × per day	1. Anti-tumor effects 2. QoL	1. Tumor shrinkage in some cases 2. Improvement in general condition and positive effects on clinical symptoms
Schütz [62]	Case reports	*N* = 23	Extra-cranial		KD with as little CHO as possible (estimated < 50 g per day), combined with insulin administration 3 × per day	1. anti-tumor effect 2. QoL	1. no anti-tumor effects found 2. reduced pain severity, but also fatigue and deteriorated orientation

*KD* ketogenic diet, *CHO* carbohydrate, *TKTL 1* transketolase-like-1, *RT* radiotherapy, *RCT* radio-chemotherapy, *QoL* quality of live, *FM* fat mass, *FFM* fat free mass, *MCT* medium-chain triglyceride, *MKD* modified ketogenic diet, *GHS* global health status, *PFS* progression-free survival, *MAD* modified Atkins diet, *DLT* dose-limiting toxicity, *TPN* total parenteral nutrition, *ERKD* restricted ketogenic diets, *FDG-PET* [8F]-2-fluoro-2–deoxy-d-glucose positron emission tomography, *HER2* human epidermal growth factor receptor 2

### Survival and disease progression

#### Results from RCTs and CTs

Overall survival was only analyzed in one RCT [23]. In this study, the overall survival (OS) for a subgroup of patients with neoadjuvant treatment for breast cancer was significantly higher in the intervention group (*p* = 0.04). However, no data for the entire study population are presented, which also consisted of patients with metastatic disease.

One RCT assessed the effects of the diet on prostate-specific antigen doubling time (PSADT) as a surrogate parameter for progression of disease [24]. Per protocol, there was no between-group difference concerning the PSADT (*p* = 0.446). Only in post hoc exploratory analysis with adjusting for multiple baseline covariates and proposed hemoconcentration, a significantly increased PSADT could be found.

#### Results from single-arm studies and case reports

Only five of these studies compared reported and expected survival, which was derived from historical controls [25–29]. In one study [25], two of the patients were analyzed and their survival was comparable with the expected survival, similar to another study where all of the different subgroups of patients had an OS in line with the historical controls [28]. Two other studies [26, 27] found a numerically better than expected survival. However, no statistical analysis was performed. One study, however, reported a lower-than-expected survival for the patients receiving a KD [29].

Another study compared the subgroup of patients, who received bevacizumab salvage treatment while on a KD with other patients treated with bevacizumab in the same hospital, who did not receive a KD. There was no difference in median progression-free survival (PFS) (*p* = 0.38) [30].

Even though most studies reported on tumor stability and progression, the results were highly heterogeneous and the tools and methods used for this assessment were only reported in a minority of them in adequate detail. Furthermore, there was no analysis for statistical significance of the findings.

An exception is the study of Fine et al. [31], which reported that patients with stable disease or partial remission on PET scan after the diet exhibited significantly higher dietary ketosis than those with progressive disease (*n* = 4, *p* = 0.018).

### Feasibility and adherence

#### Results from RCTs and CTs

Out of the included 322 patients, which were included in the 5 studies 72 drop-outs occurred (24.7%). From the 72 drop-outs, 38 (53%) were part of the intervention group and 34 (47%) of the control group [23, 24, 32–34].

#### Results from single-arm studies and case reports

Feasibility and diet adherence was analyzed in 13 studies. In total, 84 out of 139 patients (60%) were able to continue the diet for the duration of the intervention [21, 25, 28–31, 35–40].

### Quality of life

Cohen et al. used the physical component summary (PCS) and mental component summary (MCS) out of the Short Form (12) Health Survey (SF12) questionnaire to measure the quality of life (QoL) and functioning of the patients. After adjusting for baseline values and chemotherapy score, the PCS score was significantly better in the KD group. There were no significant between-group differences concerning the MCS score [32].

QoL was measured in 4 studies using the EORTC QLQ-C30 questionnaire [29, 36, 37, 41]. The results were overall inconsistent, but most often reporting stable or decreasing QoL [29, 36, 41].

### Changes in body weight

#### Results from RCTs and CTs

All 3 RCTs reported a significant higher weight loss in the KD group than in the control group [23, 24, 42]. Freedland et al. [24] found a weight loss of 12.1 kg in the intervention group, compared to a weight loss of 0.5 kg in the control group (*p* < 0.001) during the 6 months of the diet. The study of Khodabakhshi et al. [23] reported a significantly larger weight loss in the intervention group than in the control group over the course of a 3 month diet with 6.3 kg compared to 1.3 kg, respectively (*p* < 0.001). Over the same 3-month duration Cohen et al. [42] detected a weight loss of 6.1 kg in the intervention group and 3 kg in the control group (*p* < 0.05).

In one of the controlled trials by Ok et al. [34], there were no significant differences in the reduction of body weight between both groups (*p* = 0.475). In the other trial by Klement et al. [33], only regression coefficients for the changes in body weight were provided. Here, a significantly higher reduction of body weight was reported for the subgroup of breast cancer patients (*p* = 0.00014) and rectal cancer patients (*p* = 0.01). However, in the subgroup of HNC (head and neck cancer) patients the regression coefficient for “Time × KD” implied a significant positive effect of the KD on the body weight of the patients (*p* = 0.008) [33].

#### Results from single-arm studies and case reports

Changes in bodyweight were analyzed in 15 studies. A statistical analysis to check for significance was performed in 8 studies, of which 4 found a significant reduction in bodyweight [30, 36, 37, 41]. Three of these studies found a non-significant decrease in body weight [25, 31, 43] and only Fearon et al. [44] showed a significant increase in body weight.

Out of the remaining seven studies, where no statistical analysis was performed, weight loss during the diet occurred in six studies [38, 39, 45–47], while only one study showed an increase in body weight [48].

One study assessed the change in BMI and reported a median decline of 1.04 kg/m^2^, without checking for significance [28].

### Changes in body composition

#### Results from RCTs and CTs

Changes in body composition were analyzed in one RCT and both CTs. The RCT by Cohen et al. found a significant higher reduction of total fat mass in the KD group (− 5.2 kg) than in the control group (− 2.9 kg), while no significant differences concerning the lean body mass occurred [42].

Klement et al. provided primarily the regression coefficients for the fat mass (FM) and fat-free mass (FFM). In the subgroup of rectal cancer patients, a significantly greater loss of FM occurred in the KD group, without significant differences in FFM. A comparable result was reported in the subgroup of breast cancer patients, who experienced a significant reduction in FM, while the FFM reduction was not significant. However, the 50 kHz phase angle, an indicator for changes in cell mass, also significantly declined in the KD group. In the subgroup of HNC patients, the regression coefficients implied a significant increase in FFM in patients receiving a KD [33].

Ok et al. found a significantly lower reduction in body cell mass in the KD group (− 1.9 kg) than in the control group (− 2.9 kg), while no significant differences in body fat mass occurred [34].

#### Results from single-arm studies and case reports

Two studies analyzed changes in body composition [35, 41]. One study showed a significant FM reduction, without significant reduction in FFM [41], whereas the other study showed no significant effects on body composition [35].

### Adverse events

#### Results from RCTs and CTs

Only one RCT [24] and one CT [34] monitored adverse events and only the CT by Ok et al. used a validated tool [34]. In the RCT by Freedland et al. [24], only mild AEs and one moderate AE (nausea) were reported. The number of AEs was similar at baseline but increased drastically in the KD group (30 vs 19 reported AEs) at 3 months. At 6 months, the number of AEs had subsided back to baseline in the KD group and was again close to the number in the control group at the same time.

Ok et al. [34] assessed the number of meal intake-related problems and postoperative complications. No significant differences between both groups in either of the two categories occurred.

#### Results from single-arm studies and case reports

Adverse events were monitored in 19 studies. A validated tool was used in 11 of the 19 studies [26–29, 31, 35, 37, 38, 45, 46]. Since many studies combined KD with standard of care (SoC) chemotherapy and/or radiation therapy, it was often not possible to determine the cause of the reported AEs. Most of the AEs were mild to moderate. The most common AEs include: fatigue [31, 45], constipation [29, 31], diarrhea [29, 35] as well as nausea and vomiting [29, 35]. Further reported AEs were: deep venous thrombosis, asymptomatic hypoglycemia, nephrolithiasis, leg cramps, dyspepsia, dry mouth, hyperuricemia, hyperlipidemia, pedal edema, anemia, neutropenia and febrile neutropenia, thrombocytopenia, halitosis, pruritus, hypoglycemia, hyperkalemia, hypokalemia, hypomagnesemia, flu-like symptoms, low carnitine, hallucinations, allergic reaction, wound infection, headaches and neuropathy [26–31, 35–39, 45–50].

Even though most AEs were mild to moderate, there were also DLTs (dose-limiting toxicity) like CTCAE (NCI Common Terminology Criteria for Adverse Events) grade 3 dehydration, grade 4 hyperuricemia [38] and a case of grade 5 neutropenia, resulting in the death of the patient [26].

## Discussion

### Summary of main results

The basic idea of using a KD to prohibit cancer growth relies on the Warburg hypothesis and successful animal and cell culture studies. However, clinical evidence demonstrating a beneficent effect on survival and anti-tumor efficiency is still lacking.

The RCT conducted by Freedland et al. [24] failed to detect a significant anti-tumor effect in per-protocol analysis and an effect was only visible in a strongly adjusted exploratory analysis. Only Khodabakhshi et al. [23] found a significantly longer OS of the neoadjuvant treated subgroup of breast cancer patients. But said data are only presented as a Kaplan–Meier plot, without any further information, despite a *p* value of 0.04 and the claim of a higher survival rate in the KD group. It is also noteworthy that the follow-up time in this study’s Kaplan–Meier plot appears to be 26 months. However, the recruitment started in 07/2017 and stopped in 10/2018. The finished article was received by the publishing journal in 02/2019. This is just 4 months after the last patient was recruited. The resulting follow-up is 19 months at most for the first patients recruited. Furthermore, the KD was only administered for 3 months. These inconsistencies raise serious concerns regarding the presented data. Additionally, no data were provided for the subgroup of metastatic patients in this publication. Even though the data from these patients were not published as an original publication, they are reported in a systematic review by Klement et al. [51]. Here, the patients in the KD group had a numerically shorter OS (*p* = 0.078).

The studies in this review showed an overall low adherence to the KD, but the drop-out rates varied greatly between studies. Important reasons for low adherence were: limitations in monitoring and delivery [39], patients finding the meals unpalatable [38] and problems trying to integrate the diet into family life [36].

QoL was only assessed in a few studies. The RCT by Cohen et al. [32] was only able to show a significant improvement in perceived physical functioning after adjusting for several variables and without adjusting for weight loss, which attenuated the effect. No beneficial effects on mental functioning were found in this trial. This is in line with other studies, which also failed to show a QoL benefit of the KD [29, 36].

Almost all controlled and non-controlled studies showed a weight loss during the KD, which was often significant, if statistical analysis was performed [23, 24, 42]. This is rather concerning, since malnourishment, sarcopenia and cancer cachexia have been shown to negatively impact clinical outcomes and greatly reduce QoL [52, 53]. For patients with an increased risk of cancer cachexia, a KD can therefore be detrimental and the idea of implementing a KD in these patients should raise serious safety concerns. Nevertheless, studies analyzing body composition revealed that the loss of fat mass appears to be more pronounced than the loss in fat-free mass [41, 42].

The studies in this review showed a variety of adverse events related to a KD. The most frequent were fatigue [31, 45], constipation [29, 31], diarrhea [29, 35] as well as nausea and vomiting [29, 35]. Despite the fact that most of these were only mild to moderate several serious AEs like grade 3 dehydration and grade 4 hyperuricemia [38] and a case of grade 5 neutropenia occurred [26]. Especially problematic is, that many studies did not measure AEs and the ones that did, often attributed those that happened entirely to the SoC anti-cancer treatments [26]. Thus, the AEs of a KD seem to be underreported.

Finally, it should be noted that definite conclusions are still difficult to ascertain from the available data, due to a high level of bias in most studies, a small number of patients with high level of adherence and the lack of a control group and randomization, further increasing especially allocation, and performance bias. It should also be noted that in several studies the authors had a potential conflict of interest, due to financial and non-financial support or owning shares from companies producing products used in a KD [30, 35, 49, 54, 55].

Furthermore, the studies are highly heterogenous, in many cases not limited to one cancer type and often use the KD complementary to other therapies, limiting the possibility to assess whether effects and AEs were caused by the diet or other simultaneous interventions—this also impairs the possibility to pool the results to perform a meta-analysis.

### Limitations of this work

Some limitations of this systematic review must be mentioned. For once, due to the heterogeneity of the included RCTs no meta-analysis could be conducted, and no moderators of the effects caused by a KD could be determined. Furthermore, only studies published in English or German were included in this review.

## Conclusion

Even though a variety of studies have been conducted in the past on KDs for cancer patients, evidence for increased survival, anti-tumor efficacy and a reduction of side effects is lacking, even in the most recent controlled trials. More robust and consistent clinical evidence from larger patient groups with comparable methodology, thorough dietary protocols and an assessment of side effects using validated tools are necessary, before a KD can be recommended to most cancer patients. Currently possible side effects including weight loss as well as patient co-morbidities must be carefully weighed when considering applying a KD to cancer patients. To form a final judgment about the efficiency of a KD in Oncology, a randomized controlled trial with a well-designed control group and sufficient power to also detect evidence for absence of anti-tumor effects is necessary.

## Supplementary Information

Below is the link to the electronic supplementary material.

Supplementary file1 (PDF 199 kb)

Supplementary file2 (PDF 194 kb)

## Data Availability

The datasets generated during and/or analyzed during the current study are available from the corresponding author on reasonable request.

## Abbreviations

KD: Ketogenic diet
RCT: Randomized controlled trial
CT: Controlled trial
PSADT: Prostate-specific antigen doubling time
PFS: Progression-free survival
PCS: Physical component summary
MCS: Mental component summary
SF-12: Short Form (12) Health Survey
QoL: Quality of life
HNC: Head and neck cancer
FM: Fat mass
FFM: Fat-free mass
AE: Adverse events
SoC: Standard of care
DLT: Dose-limiting toxicity
CTCAE: NCI Common Terminology Criteria for Adverse Events
OS: Overall survival
