# Supplementary material for: The use of ketogenic diets in cancer patients: a systematic review
Source: Clin Exp Med. 2021 Apr 3;21(4):501–36. doi: 10.1007/s10238-021-00710-2 (PMC8505380; doi:10.1007/s10238-021-00710-2)
Supplement: Supplementary file 1 — Supplementary file1 (PDF 199 kb) [file 10238_2021_710_MOESM1_ESM.pdf]

Caption: Search-string ketogenic diets

OVID Medline
(exp Diet, Carbohydrate-Restricted/ OR exp Fasting/) OR ((Exp diet therapy/ OR diet.mp.) AND (Ketogen$ OR Breuss OR Budwig OR Gerson OR Atkins OR low-carb$ OR (low adj1 carb$) OR carbohydrate-restrict$ OR (carbohydrate$ adj3 restrict$) OR low-glycemic OR nil by mouth OR nil per os OR NPO OR fasting).mp.)

Cochrane
[mh "Diet, Carbohydrate-Restricted"] or [mh fasting] or (([mh "diet therapy"] OR diet) AND (Ketogen* OR Breuss OR Budwig OR Gerson OR Atkins OR low-carb* OR (low NEXT carb*) OR carbohydrate-restrict* OR (carbohydrate* NEXT restrict*) OR low-glycemic OR "nil by mouth" OR "nil per os" OR NPO OR fasting))

Ebsco PsychInfo
(DE "Diet" OR TX diet) AND TX (Ketogen* OR Breuss OR Budwig OR Gerson OR Atkins OR low-carb* OR (low N1 carb*) OR carbohydrate-restrict* OR (carbohydrate* N3 restrict*) OR low-glycemic OR "nil by mouth" OR "nil per os" OR NPO OR fasting)

OVID Embase
atkins diet/ OR exp diet restriction/OR high glycemic index diet/ OR exp ketogenic diet/ OR exp low carbohydrate diet/ OR ((exp diet / OR exp diet therapy/ OR diet.mp.) AND (Ketogen$ OR Breuss OR Budwig OR Gerson OR Atkins OR low-carb$ OR (low adj1 carb$) OR carbohydrate-restrict$ OR (carbohydrate$ adj3 restrict$) OR low-glycemic OR nil by mouth OR nil per os OR NPO OR fasting).mp.)

Ebsco CINAHL
MH "Diet, Low Carbohydrate" or MH "Diet, Ketogenic" OR MH "Fasting" OR ((MH "Diet+" OR TX diet) AND TX (Ketogen* OR Breuss OR Budwig OR Gerson OR Atkins OR low-carb* OR (low N1 carb*) OR carbohydrate-restrict* OR (carbohydrate* N3 restrict*) OR low-glycemic OR "nil by mouth" OR "nil per os" OR NPO OR fasting)

filters for cancer

OVID Medline
exp neoplasms/ or neoplasm$.mp or cancer$.mp. or tumo?r$.mp. or malignan$.mp. or oncolog$.mp. or carcinom$.mp. or leuk?emia.mp. or lymphom$.mp. or sarcom$.mp. or preneoplas$.mp. or exp Precancerous Conditions/ or precancer$.mp.

filters for study type

OVID Medline
(((comprehensive* or integrative or systematic*) adj3 (bibliographic* or review* or literature)) or (meta-analy* or metaanaly* or "research synthesis" or ((information or data) adj3 synthesis) or (data adj2 extract*))).ti,ab. or (cinahl or (cochrane adj3 trial*) or embase or medline or psyclit or (psycinfo not "psycinfo database") or pubmed or scopus or "sociological abstracts" or "web of science").ab. or ("cochrane database of systematic reviews" or evidence report technology assessment or evidence report technology assessment summary).jn. or Evidence Report: Technology Assessment*.jn. or ((review adj5 (rationale or evidence)).ti,ab. and review.pt.) or meta-analysis as topic/ or Meta-Analysis.pt.

OR

randomized controlled trial.pt. or controlled clinical trial.pt. or randomized.ti,ab. or placebo.ti,ab. or drug therapy.sh. or randomly.ti,ab. or trial.ti,ab. or groups.ti,ab.

Article title: The use of ketogenic diets in cancer patients: A systematic review

Journal name: Clinical and Experimental Medicine

Author names and affiliations:

Maximilian Römer, Klinik für Innere Medizin II, Hämatologie und Internistische Onkologie, Universitätsklinikum Jena, Am Klinikum 1, 07747 Jena, Germany, ORCID iD: 0000-0002-7069-4699

Jennifer Dörfler, MSc., Klinik für Innere Medizin II, Hämatologie und Internistische Onkologie, Universitätsklinikum Jena, Am Klinikum 1, 07747 Jena, Germany, ORCID iD: 0000-0003-2126-6919

Prof. Dr. med. Jutta Huebner, Klinik für Innere Medizin II, Hämatologie und Internistische Onkologie, Universitätsklinikum Jena, Am Klinikum 1, 07747 Jena, Germany

Corresponding author: Maximilian Römer, Klinik für Innere Medizin II, Hämatologie und Internistische Onkologie, Universitätsklinikum Jena, Am Klinikum 1, 07747 Jena, Germany, E-Mail: maximilian.roemer@uni-jena.de, Tel.: 0049-3641-9324256, Fax: 0049-3641-9324217
