# Supplementary material for: The use of ketogenic diets in cancer patients: a systematic review
Source: Clin Exp Med. 2021 Apr 3;21(4):501–36. doi: 10.1007/s10238-021-00710-2 (PMC8505380; doi:10.1007/s10238-021-00710-2)
Supplement: Supplementary file 2 — Supplementary file2 (PDF 194 kb) [file 10238_2021_710_MOESM2_ESM.pdf]

**Table caption:** studies excluded after full-text screening

| Excluded studies | Reason for exclusion |
| --- | --- |
| Safdie et al. 2009 [1] | Excluded since it did not meet the inclusion criteria concerning the intervention. (different fasting regimen including, but not limited to KD were used) |
| Furukawa et al. 2018 [2] | Excluded since only the abstract was published in English, while full text was only available in Japanese, therefore not meeting the Language restrictions. |
| Chu-Shore and Thiele 2010 [3] | Excluded because it reported only on patients with tuberous sclerosis complex and therefore did not meet the patient specific inclusion criteria. |

References

1. Safdie FM, Dorff T, Quinn D et al. Fasting and cancer treatment in humans: A case series report. Aging (Albany NY). 2009;1(12):988-1007. doi:10.18632/aging.100114.
2. Furukawa K, Shigematus K, Iwase Y et al. Clinical effects of one year of chemotherapy with a modified medium-chain triglyceride ketogenic diet on the recurrence of stage IV colon cancer. Journal of Clinical Oncology. 2018;36(15_suppl):e15709-e. doi:10.1200/JCO.2018.36.15_suppl.e15709.
3. Chu-Shore CJ, Thiele EA. Tumor growth in patients with tuberous sclerosis complex on the ketogenic diet. Brain Dev. 2010;32(4):318-22. doi:10.1016/j.braindev.2009.04.009.

**Article title:** The use of ketogenic diets in cancer patients: A systematic review

**Journal name:** Clinical and Experimental Medicine

**Author names and affiliations:**

Maximilian Römer, Klinik für Innere Medizin II, Hämatologie und Internistische Onkologie, Universitätsklinikum Jena, Am Klinikum 1, 07747 Jena, Germany, ORCID iD: 0000-0002-7069-4699

Jennifer Dörfler, MSc., Klinik für Innere Medizin II, Hämatologie und Internistische Onkologie, Universitätsklinikum Jena, Am Klinikum 1, 07747 Jena, Germany, ORCID iD: 0000-0003-2126-6919

Prof. Dr. med. Jutta Huebner, Klinik für Innere Medizin II, Hämatologie und Internistische Onkologie, Universitätsklinikum Jena, Am Klinikum 1, 07747 Jena, Germany

**Corresponding author:** Maximilian Römer, Klinik für Innere Medizin II, Hämatologie und Internistische Onkologie, Universitätsklinikum Jena, Am Klinikum 1, 07747 Jena, Germany, E-Mail: maximilian.roemer@uni-jena.de, Tel.: 0049-3641-9324256, Fax: 0049-3641-9324217
